# Peptide–Protein Coassemblies into Hierarchical and Bioactive Tubular Membranes

**DOI:** 10.1021/acs.biomac.2c01095

**Published:** 2023-01-25

**Authors:** Anna Majkowska, Karla E. Inostroza-Brito, Mariel Gonzalez, Carlos Redondo-Gómez, Alistair Rice, Jose Carlos Rodriguez-Cabello, Armando E. Del Rio Hernandez, Alvaro Mata

**Affiliations:** † William Harvey Research Institute, Queen Mary University of London, London EC1M 6BQ, U.K.; ‡ Institute of Bioengineering, Queen Mary University of London, London E1 4NS, U.K.; § School of Engineering and Materials Science, Queen Mary University of London, London E1 4NS, U.K.; ∥ Department of Bioengineering, Imperial College London, London SW7 2AZ, U.K.; ⊥ BIOFORGE Group, University of Valladolid - CIBER BBN, CIBER-BBN, 47011 Valladolid, Spain; # School of Pharmacy, University of Nottingham, Nottingham NG7 2RD, U.K.; ∇ Biodiscovery Institute, University of Nottingham, Nottingham NG7 2RD, U.K.; ○ Department of Chemical and Environmental Engineering, University of Nottingham, Nottingham NG7 2RD, U.K.

## Abstract

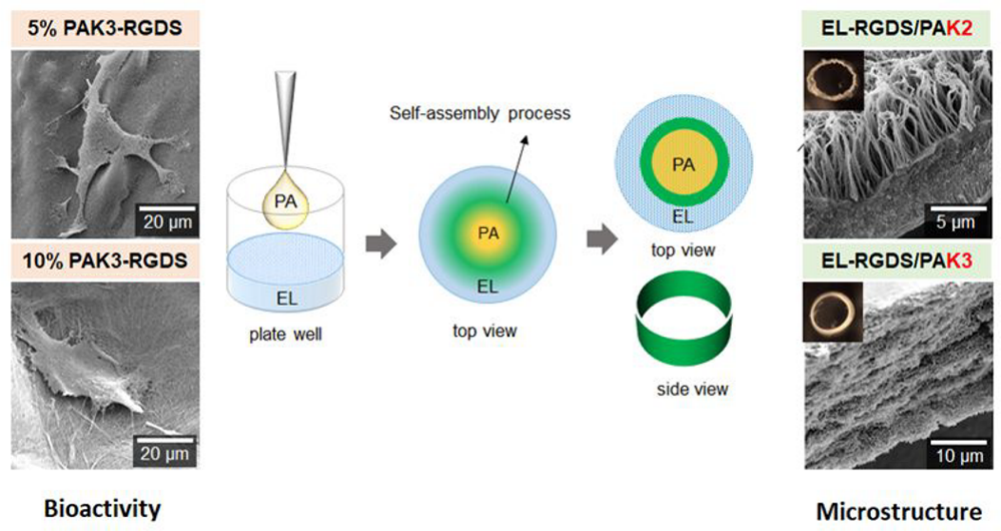

Multicomponent self-assembly offers opportunities for the design of complex and functional biomaterials with tunable properties. Here, we demonstrate how minor modifications in the molecular structures of peptide amphiphiles (PAs) and elastin-like recombinamers (ELs) can be used to generate coassembling tubular membranes with distinct structures, properties, and bioactivity. First, by introducing minor modifications in the charge density of PA molecules (PAK2, PAK3, PAK4), different diffusion-reaction processes can be triggered, resulting in distinct membrane microstructures. Second, by combining different types of these PAs prior to their coassembly with ELs, further modifications can be achieved, tuning the structures and properties of the tubular membranes. Finally, by introducing the cell adhesive peptide RGDS in either the PA or EL molecules, it is possible to harness the different diffusion-reaction processes to generate tubular membranes with distinct bioactivities. The study demonstrates the possibility to trigger and achieve minor but crucial differences in coassembling processes and tune material structure and bioactivity. The study demonstrates the possibility to use minor, yet crucial, differences in coassembling processes to tune material structure and bioactivity.

## Introduction

Self-assembly is ubiquitous in nature, enabling molecules to spontaneously interact and organize into higher ordered structures. For instance, the functionality of proteins depends on the unique amino acid sequences at the molecular level as well as on protein–protein interactions at the supramolecular scale. Consequently, there has been a growing interest in using self-assembly to engineer functional materials using simple peptide building-blocks.^1,2^ Examples include short diphenylalanine-containing peptides that assemble into stable^3^ and robust micron-long nanotubes^4^ and tyrosine-containing tripeptides that assemble into nanofibrous structures encoding controlled modifications in UV absorbance, coloration, and electrochemical properties.^5^

Peptide amphiphiles (PAs), developed by Stupp and colleagues,^6^ are an attractive family of self-assembling peptides capable of generating nanofibrous matrices displaying a plethora of functionalities through surface-displaying peptides. Examples include PA nanofibers mimicking glycosaminoglycans to induce regeneration of cardiovascular tissue,^7^ displaying dynamic cell binding epitopes,^8^ or adaptive signals capable of promoting spinal cord regeneration.^9^ The versatility of these molecules also enables structural modifications by incorporating matrix metalloproteinase cleavage sites in order to reveal a hidden bioactive region after controlled degradation,^10^ integrating host–guest moieties to tailor interfiber interactions,^11−13^ or enabling hierarchical assembly into aligned nanofibers.^14^ However, these systems alone remain far from recreating the diverse multiscale signaling observed in the native extracellular matrix (ECM).^15^

Multicomponent self-assembly faciliates controlled integration of multiple types of building-blocks leading to materials with properties that not only combine those of the individual components but also emergent ones.^16^ Through this approach, O’Reilly and co-workers developed a temperature-responsive bioconjugate system of superfolder green fluorescent protein (sfGFP) and poly[(oligo ethylene glycol) methyl ether methacrylate] (PEGMA).^17^ Draper et al. went a step further, developing a spatially resolved multicomponent network from low-molecular-weight hydrogels and then removing one of the networks selectively.^18^ In a similar approach, a modified 1,3:2,4-dibenzylidenesorbitol (DBS) was combined with a byproduct from its synthesis (MBS-CO_2_Me) forming a two-component supramolecular hydrogel with improved mechanical stability.^19^ Other examples include the coassembly of PAs and laponite to trigger and control hierarchical mineralization.^20^

Pioneering work by Stupp and colleagues demonstrated how coassembling PAs with hyaluronic acid (HA) can trigger compartmentalization and controlled diffusion leading to the hierarchical assembly of PA-HA structures.^21^ Inspired by this work, we have developed coassembling platforms that take advantage of protein disorder-to-order transitions to enhance stability,^22−24^ tailor mechanical properties,^25^ and engineer bioactive environments.^26−29^ In particular, by coassembling PAs with elastin-like recombinamers (ELs), we have demonstrated the possibility to modulate EL conformation at liquid–liquid interfaces to grow PA-EL tubular constructs with hierarchical structure.^30^ The ability to fabricate intricate tubular membranes in an easy one-step self-assembling process offers advantages for applications in tissue engineering. First, tubular architectures such as those in the vascular, lymphatic, and gut systems are common and critical for organisms. Second, the capacity to fabricate these tubular structures displaying thin, soft, permeable, and hierarchical materials opens opportunities to better recreate physiological properties compared to other synthetic materials commonly used in microfluidic devices. Third, the interfacial self-assembly process enables incorporation and localization of additional components to enhance compositional and structural complexity.^31^ Altogether, these features demonstrate the potential opportunities that self-assembling tubular structures could offer.

Here, we report on the possibility to generate protein-peptide coassembling materials with tunable structure and bioactivity. We use the EL/PA system and demonstrate how minor structural modifications in the amino acid sequences of ELs and PAs can tailor the mechanism of assembly and lead to reproducible structural changes in the coassembled materials. Furthermore, by introducing cell binding RDGS sequences in the PA molecules, we fine-tune the bioactivity of these constructs. We characterize fiber organization, material (i.e., membrane) thickness, and speed of material degradation as well as bioactivity including cell adhesion, morphology, and metabolic activity.

## Experimental Section (Materials & Methods)

### Tubular Membrane Formation

EL and PA molecules were dissolved separately in Milli-Q water (10 and 15 mg/mL respectively). EL solutions were reconstituted and then incubated at 4 °C for 15 min to allow proper dissolution of molecules. For PAK3/PAK3-RGDS: mixtures containing different percentages of RGDS motif were prepared by mixing the PAK3 filler solution with different volumes of PAK3-RGDS solution until a 5, 10, 20, 50, or 100% (w/v) concentration of PAK3-RGDS was reached and the total peptide concentration was kept at 1.5% (w/v). For PA mixtures: both PAs were first dissolved separately and then mixed in 1:1 ratio to form PAK2-K4, PAK2-K3, and PAK3-K4 mixtures. pH was adjusted to pH = 5 (EL) and pH = 4.5 (PA). The pH values were chosen based on the previous studies^28,29^ as optimal for formation of a robust membrane. A 190 μL aliquot of EL solution was placed in a well in a 48 well plate. A 10 μL aliquot of a PA mixture solution was then added by immersing the pipet tip into the EL solution and slowly releasing the liquid. The tubular membrane was left to coassemble for 48 h at RT. The resultant structure remained attached on the bottom to the well plate. The aqueous solution was removed from the sample well (without touching the membrane), and several rinses with Milli-Q water were performed to remove any debris from the coassembly process.

### Stability Studies of ELs/PAK3/PAK3-RGDS Tubular Membranes

Tubular membranes were prepared as described above. Then, membranes were washed in Milli-Q water and cross-linked with glutaraldehyde (4% (w/v)) for 2 h at RT, followed by washing in Milli-Q water. Tubular membranes were then submerged in PBS (1×) solution at RT. Bright-field images were collected after membrane formation, after cross-linking, immediately after submerging in PBS (1×) solution, and then every 7 days for up to 4 weeks.

### Stability Studies – TNBSA Assay

Tubular membranes were prepared as described previously. After coassembling for 48 h at RT, tubular membranes were washed three times with Milli-Q water and covered with PBS (1×) solution. A volume of solutions in which tubular membranes were submerged was collected at each of the investigated time points: 5 and 60 min, 6 and 24 h, and 7 days. A 25 μL aliquot of sample was placed in a well plate and topped up with 75 μL of PBS 1× and 50 μL of 0.01% 2,4,6-trinitrobenzenesulfonic acid (TNBSA) in PBS. Plates were incubated at 37 °C for 2 h. Then, the reaction was stopped by addition of 50 μL of 10% SDS and 25 μL of HCl 1 M. A standard curve was prepared by dissolving glycine in a series of known concentrations. Absorbance was measured at 335 nm at RT using a microplate reader (Spetrostarnano, BMG Labtech, UK). Measurements were conducted in duplicates and repeated twice.

### Scanning Electron Microscopy

EL/PA tubular membranes were left to coassemble for 48 h, washed in Milli-Q water, and fixed with 2.5% glutaraldehyde in Milli-Q water for 2 h at RT. Then, the samples were washed in Milli-Q water followed by dehydration in increasing concentrations of ethanol (20, 50, 70, 90, 96, and 100%) while still remaining attached to the bottom of the well plate. After the dehydration step, the samples were carefully removed from the well plate using tweezers and transferred to the critical point dryer holder. The samples were then dried in a process of critical point drying (K850, Quorum Technologies, UK). Dried samples were attached to the SEM stubs using carbon tape and manipulated with tweezers and scalpel in order to reveal their cross-sectional area. Then, samples were sputter-coated with gold for 90 s. SEM imaging was carried out using an Inspect F50 (FEI Comp, The Netherlands).

### Atomic Force Microscopy

Atomic force microscopy was used to measure the Young’s moduli of different tubular membranes fabricated in the study. Tubular membranes were cut open with a scalpel, transferred with tweezers to a new Petri dish, and placed on either the luminal or abluminal side of the membrane facing downward. In this way, the measurements could be conducted on either side of the membrane. The samples were then attached to a Petri dish using a drop of cyanoacrylate adhesive followed by immersion in ultrapure Milli-Q water. Young’s modulus measurements were taken with JPK Nanowizard-1 (JPK Instruments, Germany) in force spectroscopy mode, which was mounted on an inverted optical microscope (IX-81, Olympus, Japan). Indentation was carried out using quadratic pyramidal cantilevers (MLCT, Bruker, MA, USA) with a spring constant of 0.07 N/m and a half angle to face of 17.5°. The sensitivity of the cantilevers was first assessed by analyzing the gradient of the force–distance curve in the JPK Nanowizard-1 software on an empty region of a Petri dish. This was then followed by sample indentation with an approach speed of 5 μm/s and maximum set force of 1 nN. Five independently fabricated membranes were prepared for each condition. Measurements were taken across at least 5 regions of 100 × 100 μm^2^ size per sample and at least 5 times per area yielding 25 measurements per condition. Young’s moduli were calculated by fitting the contact region of the acquired force curves with the Hertz Contact Model using the JPK software, the above constants, and calibrated cantilever sensitivity.

### Cell Studies

Fully coassembled tubular membranes were washed with Milli-Q water and cross-linked with genipin at concentration of 25 μL/mL at 37 °C overnight. Tubular membranes were then washed in Milli-Q water and sterilized under UV light for 20 min. After sterilization, tubular membranes were washed three times in Hank’s balanced salt solution. A total of 50 000 mADSCs resuspended in DMEM (20% FBS, 1% P/S) were seeded on each EL/PA tubular membrane, while they were still attached to the bottom of the well plate in a vertical position. The constructs were then agitated for 30 min at 150 rpm before culture in static conditions to allow for uniform cell attachment. Media was changed every 2 to 3 days.

### Cell Adhesion

Cells were seeded as previously described in serum-free DMEM media and incubated for 4 h followed by an additional 20 h in full media (DMEM supplemented with 20% FBS). Cells were fixed with 4% paraformaldehyde for 1 h and stained with blue dye 4′-6-diamino-2-phenylindole (DAPI). After staining, membranes were carefully removed from the well plate with tweezers, cut open with a scalpel, and placed on the microscope slide followed by imaging under an epifluorescent microscope (Leica DMi8).

Cell metabolic activity was assessed on days 2, 7, and 14 with an Alamar Blue cell metabolic assay. Tubular membranes were incubated for 2 h at 37 °C in a 10% (v/v) solution of Alamar Blue in DMEM. Fluorescence of the solution was then read at 570 and 595 nm using a microplate reader (Spetrostarnano, BMG Labtech, UK).

Cell proliferation was assessed by quantifying the number of adherent cells to tubular membranes with Quant-iT PicoGreen assay on days 2, 7, and 14. Briefly, cells were lysed, and the supernatant solution was diluted in assay buffer followed by addition of Quant-iT PicoGreen reagent and incubation for 5 min at RT. Fluorescence of the samples was measured at 480 nm (excitation) and 520 nm (emission) using a microplate reader (Spetrostarnano, BMG Labtech, UK). The DNA concentration for each sample was calculated by using a standard curve.

## Results and Discussion

### Rationale

**Figure 1:**
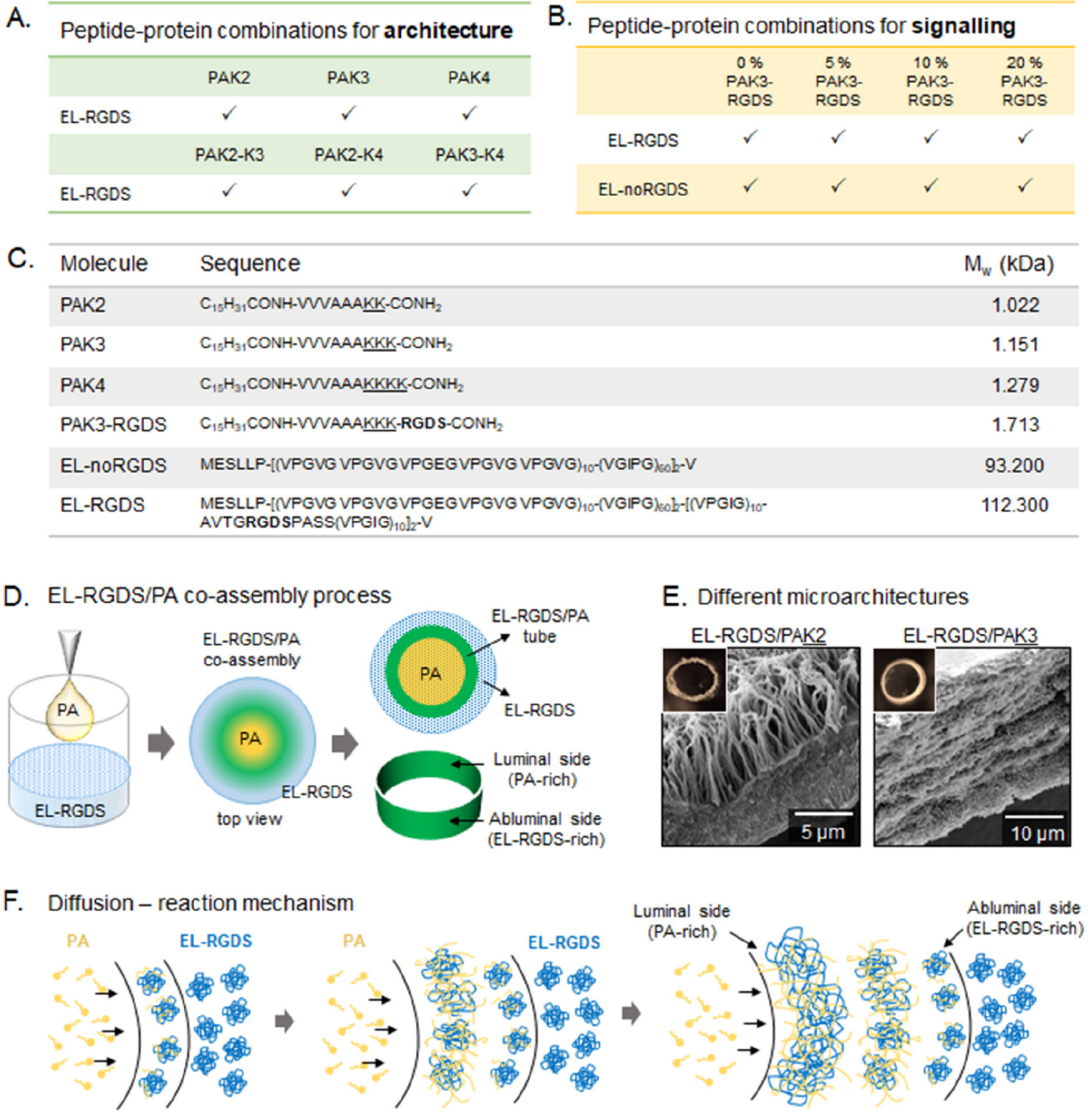
Experimental design and schematic representation of tubular membranes. (A) Peptide and protein combinations used for modification of architecture. (B) Peptide and protein combinations used for modification of signaling. (C) Molecular structure information for PA and EL molecules used in this study. (D) Schematic diagram of the process of EL/PA coassembly and tubular shape of the resulting structure. (E) SEM micrographs showing different types of EL/PA microstructures. (F) Schematic diagram of the diffusion-reaction mechanism.

We aim to modulate EL/PA material bioactivity and architecture through minor structural modifications in the PA and EL molecules (Figure 1C,D). We use three PA molecules (PAK2, PAK3, PAK4) varying in charge density depending on the number of lysines (K) present in their structures (Figure 1C). We have previously used these molecules to coassemble into tubular structures and demonstrated that their individual amino acid sequences can have profound effects in their coassembly mechanism and material properties^30^ (Figure 1E). Consequently, here we investigate how different PAs and their mixtures (PAK2-PAK3, PAK2-PAK4, and PAK3-PAK4) can modify microstructure and mechanical properties of the resulting EL/PA tubular membranes. With the aim of modifying bioactivity, we test PAs and ELs with (PAK3-RGDS, EL-RGDS) and without (PAK3, EL-noRGDS) the cell binding peptide RGDS. By coassembling PA molecules decorated with RGDS epitope (PAK3-RGDS) with a diluent molecule (PAK3), we can control spacing for optimal cell recognition, as previously reported for PA materials.^32^ To dissect the effect of the PAK3-RGDS molecules, ELs (EL-noRGDS) are coassembled with bioactive PAs (PAK3-RGDS), resulting in tubular membranes (EL-noRGDS/PAK3/PAK3-RGDS) where the only source of RGDS is the PAK3-RGDS molecules. The resulting materials are characterized according to their mechanical properties and used as cell culture substrates to investigate their effect on cell adhesion, proliferation, metabolic activity, and morphology.

### Synthesis of Individual Components and Tubular Membrane Fabrication

PA molecules were synthesized following standard solid-phase peptide synthesis methods as previously reported.^33^ Proof of PA purity and structural conformation were acquired through reverse phase HPLC and electrospray ionization mass spectrometry as detailed in the Supporting Information (Figure S1). PAK3-RGDS molecules were obtained from Cambridge Peptides (Birmingham, UK), while ELs were obtained from Technical Proteins Nanobiotechnology S. L. (Valladolid, Spain). EL/PA tubular membranes were fabricated as previously reported.^30^ Briefly, a small volume of PA solution (10 μL, 1.5% (w/v), pH = 4.5) was inoculated in a larger volume of EL solution (190 μL, 1% (w/v), pH = 5) and left to coassemble for 48 h at room temperature (RT) (Figure 1D). The resulting tubular membranes exhibited a multilayered microstructure with layers formed by nanofibers composed of PAs and ELs. Due to the diffusion-based mechanism of assembly,^30^ the layers exhibited progressively different concentrations of ELs and PAs from the luminal (inner) to the abluminal (outer) side (Figure 1F). Toward the luminal side, there was a higher concentration of PAs (PA-rich side), whereas toward the abluminal side, there was a higher concentration of EL molecules (EL-rich side) (Figure 1D).

### Modification of Architecture of EL/PA Tubular Membranes

#### PA Mixtures – Tubular Membrane Macrostructure and Microarchitecture

**Figure 2:**
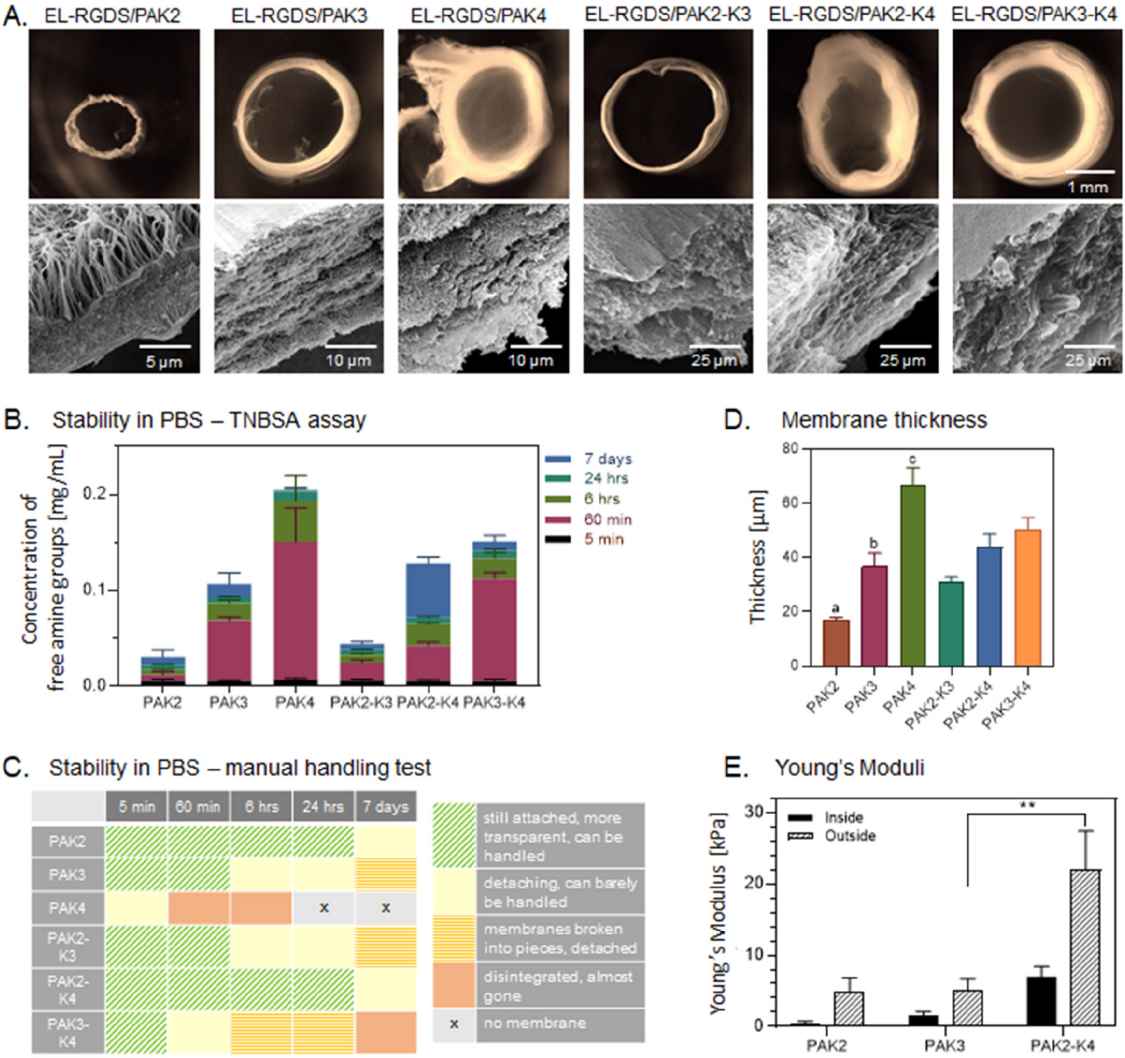
Characterization of structure and physical properties of the EL-RGDS/PA tubular membranes depending on the PA used (PAK2, PAK3, PAK4 and their mixtures: PAK2-K3, PAK2-K4, PAK3-K4). (A) Top row: bright-field micrographs of the EL-RGDS/PA tubular membranes’ top-view (the yellow color is an artifact of cross-linking with glutaraldehyde); bottom row: SEM cross-sectional micrographs of the corresponding EL-RGDS/PA tubular membrane. (B) Stability of the EL-RGDS/PA membranes in PBS (1×) solution was measured by TNBSA assay at 5 min, 60 min, 6 h, 24 h, and 7 days. Error bars represent ±SD. The experiments were performed in duplicates (*n* = 4). (C) Stability of the EL-RGDS/PA membranes in PBS (1×) as investigated by manual handling (each membrane was picked up with the tweezers, removed from the solution, and placed back in the same well in a well plate). The experiments were performed in duplicates. (D) Thickness of the membranes as measured by SEM. Error bars represent ±SD. The experiments were performed in triplicate (*n* = 9). a – PAK2 vs PAK3 *, PAK2 vs PAK4 ****, PAK2 vs PAK2/K4 **, PAK2 vs PAK3/K4 ***, b – PAK3 vs PAK4 ***, c – PAK4 vs PAK2/K3 ****, PAK4 vs PAK2/K4 *. (E) Young’s moduli of the membranes. AFM measurements were carried out on the luminal and abluminal sides of the EL-RGDS/PA membranes. Error bars represent ±SEM. Four membranes were prepared per condition, each measured in five different areas (*n* = 20). Error bars represent ±SEM where **** corresponds to *p* < 0.0001, *** corresponds to *p* < 0.001, ** corresponds to *p* < 0.01, and * corresponds to *p* < 0.05.

To modify architecture, stability, and mechanical properties of the EL/PA tubular membranes, we investigated how using mixtures of different PAs (PAK2, PAK3, and PAK4) would influence these properties (Figure 1A). EL/PA tubular membranes were manufactured as described above. Briefly, a small volume of PA mixture solution was inoculated in a larger volume of EL solution and left to coassemble for 48 h at RT resulting in formation of a tubular membrane (Figure 1D). Observations under an optical microscope revealed that depending on the PA mixture used, the resulting tubular membranes differ in diameter and thickness (Figure 2A). The smallest in diameter was the EL-RGDS/PAK2 tubular membrane, followed by EL-RGDS/PAK3, while the mixture of these two (EL-RGDS/PAK2-K3) generated a tubular membrane with a similar diameter as EL-RGDS/PAK3. The largest in diameter was EL-RGDS/PAK4, as well as both of its mixtures EL-RGDS/PAK2-K4 and EL-RGDS/PAK3-K4. As well as being the largest tubular membranes in diameter, EL-RGDS/PAK4, EL-RGDS/PAK2-K4, and EL-RGDS/PAK3-K4 exhibited thicker walls with a looser composition compared to EL-RGDS/PAK2 and EL-RGDS/PAK3, while the EL-RGDS/PAK2-K3 tubular membrane morphologically resembled EL-RGDS/PAK2 when it comes to wall thickness and tightness (Figure 2A). Further SEM observations of the cross sections of the tubular membrane wall revealed that all of the coassembled systems exhibited multilayered microarchitecture (Figure 2A) except for EL-RGDS/PAK2. This tubular membrane displayed a three-section structure with orthogonal fibers, suggesting a different mechanism of coassembly. Quantification of tubular membrane thickness from SEM cross-sectional micrographs confirmed the bright-field microscopy results indicating significant differences in thickness between all of the investigated systems (Figure 2D). These results indicate that the charge of PAs and PA mixtures used in tubular membrane formation has an immediate effect on the macro- and microproperties of the resulting structures.

#### PA Mixtures – Tubular Membrane Stability

To characterize stability of all of the investigated EL-RGDS/PA tubular membranes (EL-RGDS/PAK2, EL-RGDS/PAK3, EL-RGDS/PAK4, EL-RGDS/PAK2-K3, EL-RGDS/PAK3-K4, EL-RGDS/PAK2-K4), we conducted a TNBSA assay to measure the stability of the membranes by detecting the amount of amine groups released into the solution after submerging the tubular membranes in a phosphate buffered solution (PBS) (1×). Measurements were taken at 5 min, 60 min, 6 h, 24 h, and 7 days (Figure 2B). Additionally, manipulation tests were carried out (Figure 2C) consisting of handling the tubular membranes with tweezers from the well onto a glass slide and then handling them back to the original well. The TNBSA assay revealed that the EL-RGDS/PAK2 released the least amount of amine groups at all time points (Figure 2B), suggesting a high stability of the membranes. The high stability of the membrane was then confirmed by manipulation tests (Figure 2C). The least stable membranes were the EL-RGDS/PAK4 combination which exhibited the highest release of amine groups on the TNBSA assay (Figure 2B). However, upon mixing PAK2 with PAK4, the resulting tubular membranes (EL-RGDS/PAK2-K4) exhibited much-improved stability and exceptional handability compared to EL-RGDS/PAK4 (Figure 2B,C). A similar improvement in stability was observed when mixing PAK2 and PAK3 (EL-RGDS/PAK2-K3) compared to EL-RGDS/PAK3 tubular membranes evidenced by Figure 2B. Based on these results, we decided to carry out the mechanical testing and biocompatibility studies using the most stable membranes, EL-RGDS/PAK2, EL-RGDS/PAK3, and EL-RGDS/PAK2-K4. These results indicate that minor modifications in molecular design lead to changes in stability and degradation profiles in ionic solutions.

#### Mechanical Testing

We investigated mechanical properties of the three selected systems (EL-RGDS/PAK2, EL-RGDS/PAK3, and EL-RGDS/PAK2-K4). We measured Young’s moduli by conducting atomic force microscopy (AFM) measurements on both luminal and abluminal sides of the tubular membranes. The results revealed no significant difference in Young’s moduli between EL-RGDS/PAK2 and EL-RGDS/PAK3 systems on luminal and abluminal sides (Figure 2E). However, the Young’s modulus of EL-RGDS/PAK2-K4 tubular membranes on the abluminal side was significantly higher than that of the EL-RGDS/PAK3 tubular membrane. A similar increase was evident in the luminal side of the EL-RGDS/PAK2-K4 tubular membranes compared to both EL-RGDS/PAK2 and EL-RGDS/PAK3 tubular membranes. These differences might be caused by differences in the microarchitectural structure of the coassembling systems. These results are in agreement with the manual handling tests, indicating the EL-RGDS/PAK2-K4 tubular membranes are more robust than both EL-RGDS/PAK2 and EL-RGDS/PAK3 ones (Figure 2C).

#### Cell Adhesion

**Figure 3:**
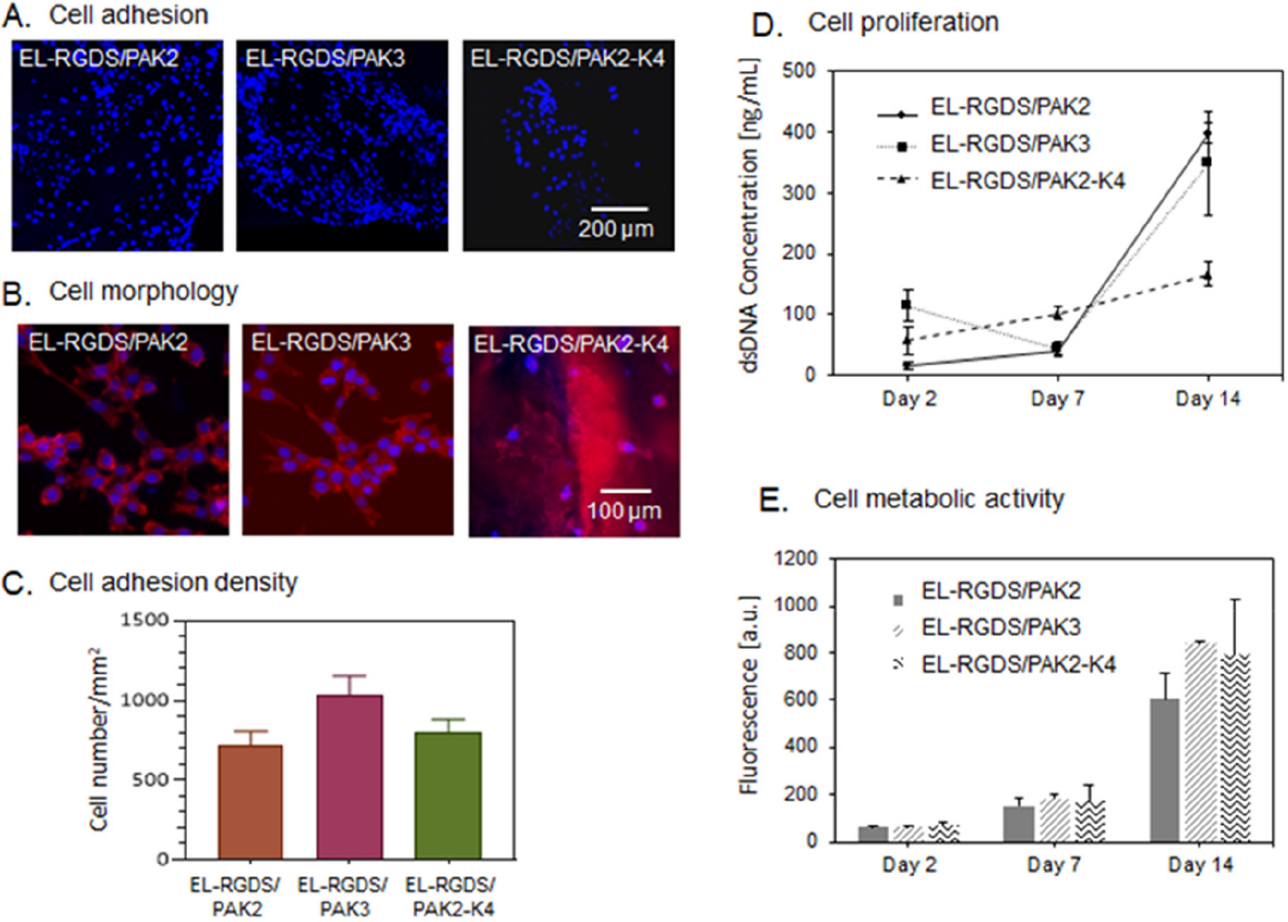
Investigation of biocompatibility of the EL-RGDS/PA membranes. Mouse-derived adipose stem cells (mADSCs) were cultured on the membranes. (A) Fluorescence microscopy images of mADSCS stained with DAPI after 24 h of cell culture. (B) Morphology study. mADSCs were stained with Phalloidin CruzFluor 647 and DAPI. Images indicate a spreading morphology and intercellular connections between mADSCS grown on both EL-RGDS/PAK2 and EL-RGDS/PAK3 membranes, in contrast to mADSCs grown on EL-RGDS/PAK2-K4, which were observed to be not spread and with minimal intercellular connections formed. (C) Cell adhesion density as calculated based on the adhesion study images stained with DAPI (A). (D) Proliferation studies. dsDNA content was quantified by PicoGreen assay. (E) Cell metabolic activity was assessed with Alamar Blue assay. Error bars represent ±SD. The experiments were performed in triplicates (*n* = 6).

We then assessed the role of PAs and PA mixtures used in this study on membrane’s biocompatibility. mADSCs were cultured on the EL-RGDS/PAK2, EL-RGDS/PAK3, and EL-RGDS/PAK2-K4 tubular membranes. Biocompatibility was assessed by quantifying cell adhesion, morphology, viability, and proliferation. mADSCs were seeded on both luminal and abluminal sides of the tubular membranes in serum-free medium, incubated for 4 h, rinsed to remove the nonadherent cells, incubated for an additional 20 h in full media (DMEM, 20% FBS), and then dyed with 4′-6-diamino-2-phenylindole (DAPI). Fluorescent microscopy indicated higher numbers of cells growing on both EL-RGDS/PAK2 and EL-RGDS/PAK3 tubular membranes compared to EL-RGDS/PAK2-K4 tubular membranes (Figure 3A). Quantitative analysis of the micrographs confirmed these findings, revealing a higher density of cells growing on EL-RGDS/PAK3 tubular membranes compared to both EL-RGDS/PAK2 and EL-RGDS/PAK2-K4 ones (Figure 3C). We hypothesize that the decrease in cellular adhesion may be the result of (i) a greater cytotoxic effect from the high positive charge of PAK4^30^ or (ii) the higher Young’s Modulus of EL-RGDS/PAK2-K4 (compared to EL-RGDS/PAK2 and EL-RGDS/PAK3 (Figure 2E)), which could influence cell adhesion. Previous studies have demonstrated that stiffer surfaces can result in lower mADSC adhesion.^34^

#### Cell Morphology

mADSCs cultured on the tubular membranes were stained with DAPI (nucleus) and AlexaFluor Phalloidin 647 (actin) and imaged under an epifluorescent microscope. Analysis of the micrographs revealed that cells grown on both EL-RGDS/PAK2 and EL-RGDS/PAK3 membranes exhibited a spread morphology with numerous intercellular connections formed (Figure 3B). Nuclei were observed to be round with a good amount of cytoplasm surrounding, which indicates a healthy cell. In contrast, cells grown on EL-RGDS/PAK2-K4 tubular membranes had much less spread morphology and formed fewer connections with the neighboring cells, suggesting the EL-RGDS/PAK2-K4 tubular membrane might be less biocompatible than EL-RGDS/PAK2 and EL-RGDS/PAK3 systems.

#### Cell Proliferation

Cell proliferation was then assessed by quantification of dsDNA concentration of mADSCs grown on different tubular membranes on days 2, 7, and 14 via a Quant-iT PicoGreen assay. Results revealed that proliferation rate of mADSCs grown on EL-RGDS/PAK2-K4 tubular membranes is much slower than proliferation rates of mADSCs grown on EL-RGDS/PAK2 and EL-RGDS/PAK3 tubular membranes (Figure 3D). At day 14, we observed an almost 2-fold increase in the concentration of dsDNA in the case of both EL-RGDS/PAK2 and EL-RGDS/PAK3 compared to EL-RGDS/PAK2-K4, suggesting that possible cytotoxicity of PAK4 may play a role. Although EL-RGDS/PAK2-K4 system supports initial cell adhesion, it may not support cell proliferation as well as the other two systems, EL-RGDS/PAK2 and EL-RGDS/PAK3.

#### Cell Metabolic Activity

In order to further assess the capacity of these materials to support cell growth, we investigated cell metabolic activity via an Alamar Blue assay over 2 weeks of culture. We observed no significant differences between all of the investigated systems at any of the time points (Figure 3E). These results suggest that although mADSCs grown on EL-RGDS/PAK2-K4 tubular membranes exhibit lower rates of proliferation, they are still as metabolically active as cells cultured on EL-RGDS/PAK2 and EL-RGDS/PAK3 tubular membranes.

These results suggest that a combination of PA and EL molecules is needed to generate the unique EL/PA tubular membranes, and by tuning the molecular structure of PA as well as the relative composition (i.e., ratio) of the PA mixture, we can obtain materials with completely different microstructures and properties, such as improved stability in an ionic environment and better mechanical properties. Use of PAs with higher charge density such as PAK4 may lead to decreased biocompatibility, therefore limiting the usefulness of these systems.

### Modification of Bioactivity of EL/PA Membranes

#### Structure and Stability of EL-RGDS/PAK3-RGDS Tubular Membranes

**Figure 4:**
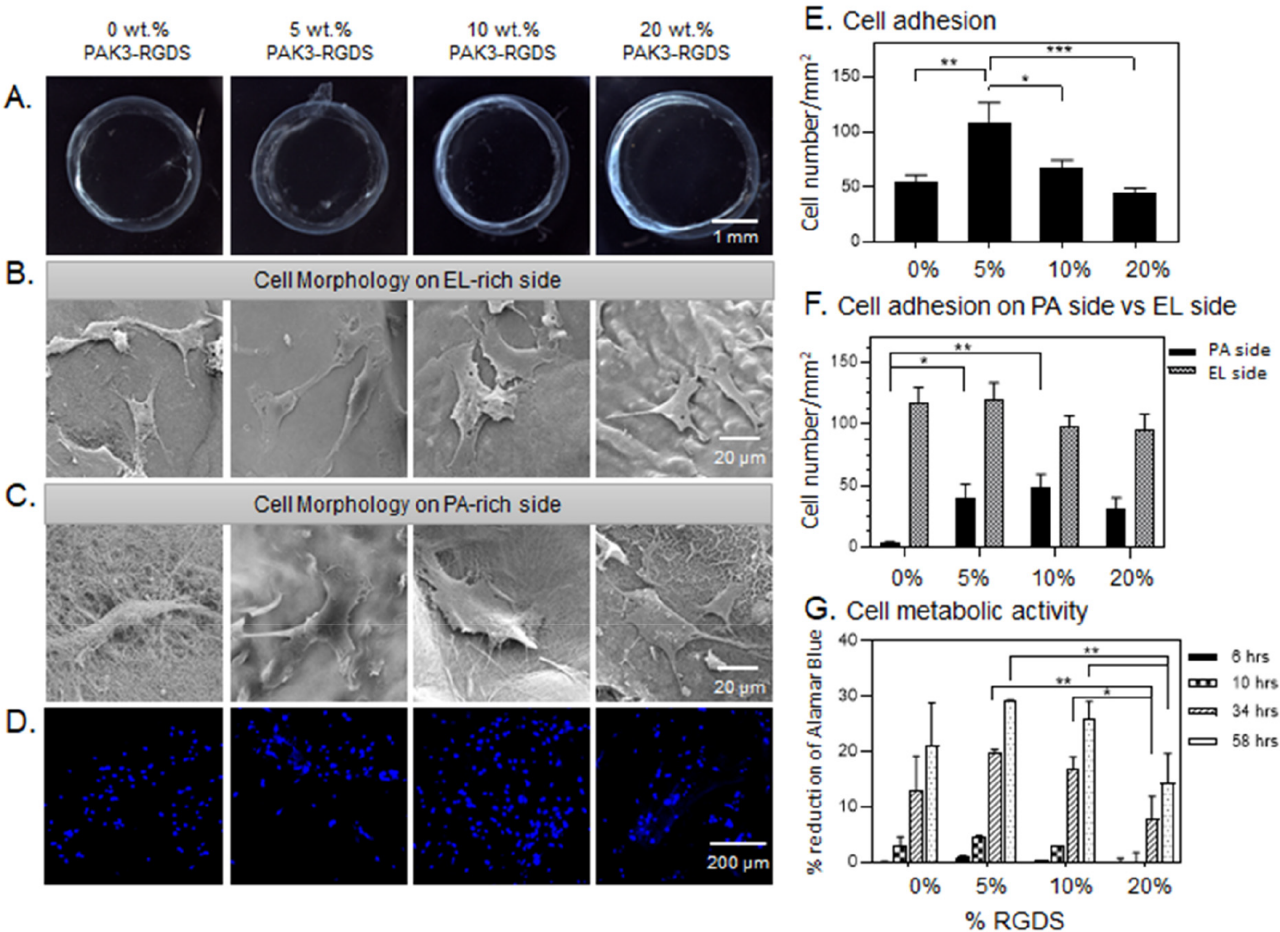
Modification of bioactivity of EL-RGDS/PAK3/PAK3-RGDS membranes. (A) Bright-field images of EL-RGDS/PAK3/PAK3-RGDS membranes depending on concentration of PAK3-RGDS (0, 5, 10, and 20% (w/v)). (B,C) Morphology study. SEM micrographs of the membranes with mADSCs attached to the abluminal side of the membrane (EL side) (B), and SEM micrographs of the membranes with cells attached to the luminal side of the membrane (PA side) (C). (D) Cell adhesion study. mADSCs were stained with DAPI followed by imaging. (E) Cell adhesion density based on analysis of confocal images in (D). Error bars represent ±SEM. (F) Cell adhesion density on the luminal side of the membrane versus abluminal side of the membrane based on analysis of SEM micrographs. Error bars represent ±SEM. (G) Cell metabolic activity as assessed with an Alamar Blue assay. Error bars represent ±SD. Each experiment was conducted in triplicates (*n* = 6). Error bars represent ± SD or ± SEM where **** corresponds to *p* < 0.0001, *** corresponds to *p* < 0.001, ** corresponds to *p* < 0.01, and * corresponds to *p* < 0.05.

**Figure 5:**
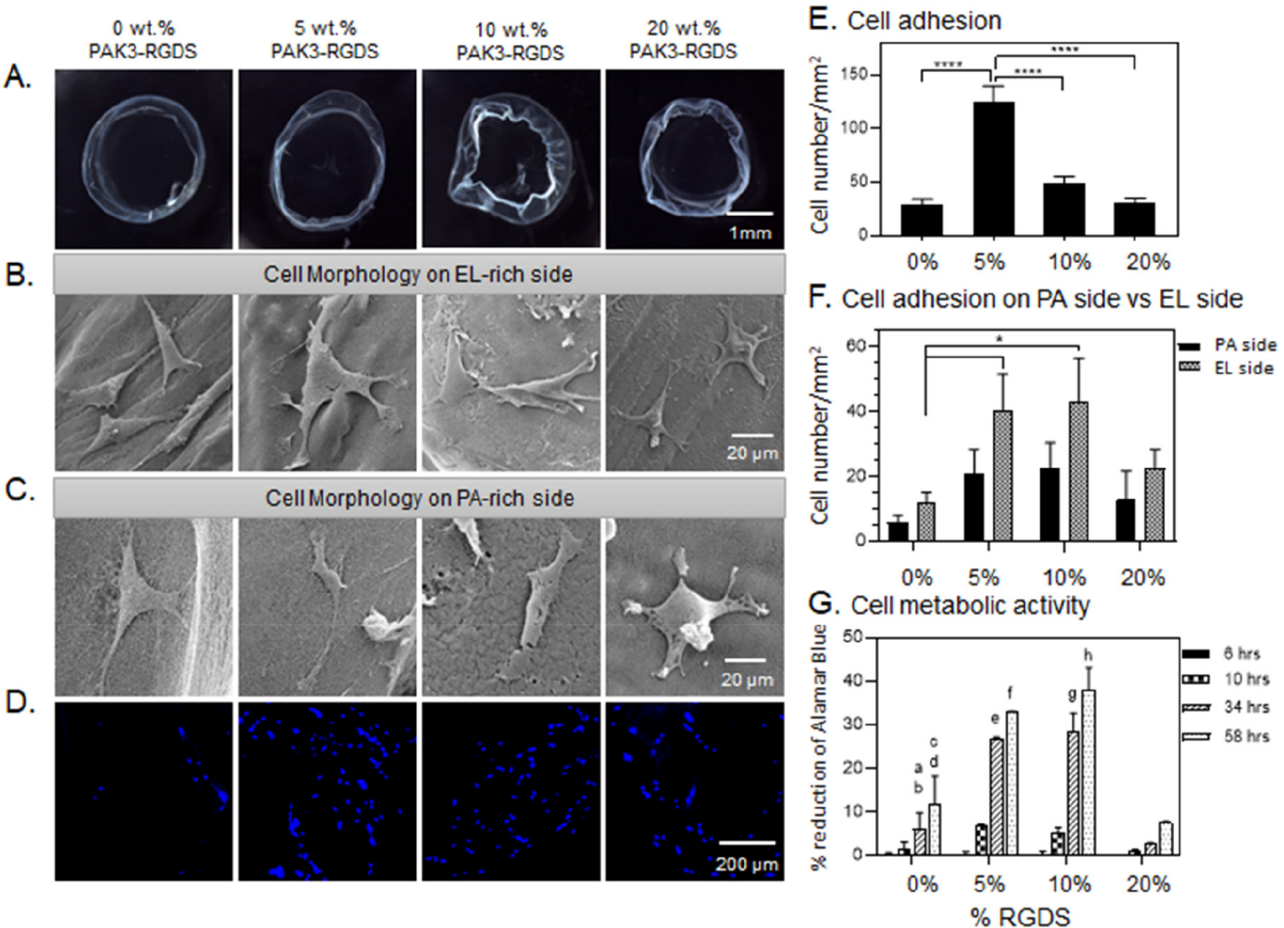
Modification of bioactivity of EL-noRGDS/PAK3/PAK3-RGDS membranes. (A) Bright-field images of EL-RGDS/PAK3/PAK3-RGDS membranes depending on concentration of PAK3-RGDS (0, 5, 10, and 20% (w/v)). (B,C) Morphology study. SEM micrographs of the membranes with mADSCs attached to the abluminal side of the membrane (EL side) (B), and SEM micrographs of the membranes with cells attached to the luminal side of the membrane (PA side) (C). (D) Cell adhesion study. mADSCs were stained with DAPI followed by imaging. (E) Cell adhesion density based on analysis of confocal images in (D). Error bars represent ±SEM. (F) Cell adhesion density on the luminal side of the membrane versus abluminal side of the membrane based on analysis of SEM micrographs. Error bars represent ±SEM. (G) Cell metabolic activity as assessed with an Alamar Blue assay. Error bars represent ±SD; a – 0 vs 5% at 34 h ****, b – 0 vs 10% at 34 h ****, c – 0 vs 5% at 58 h ****, d – 0 vs 10% at 58 h ****, e – 5 vs 20% at 34 h ****, f – 5 vs 20% at 58 h ****, g – 10 vs 20% at 34 h ****, h – 10 vs 20% at 58 h ****. Each experiment was conducted in triplicates (*n* = 6). Error bars represent ±SD or ±SEM where **** corresponds to *p* < 0.0001, *** corresponds to *p* < 0.001, ** corresponds to *p* < 0.01, and * corresponds to *p* < 0.05.

EL/PA coassembled systems are structurally sensitive to molecular modifications.^30^ For this reason, we first investigated the addition of RGDS by testing PAK3-RGDS molecules in the coassembling system (Figure 1B). We prepared PA/PA-RGDS mixtures by mixing the PAK3 filler solution with different volumes of PAK3-RGDS solution until a 5, 10, 20, 50, 70, or 100% (w/v) concentration of PAK3-RGDS was reached, while the total peptide concentration was kept at 1.5% (w/v). Tubular membranes were fabricated as described above. To investigate the stability of the tubular membranes, samples were submerged in a solution of PBS 1× at RT for up to 4 weeks. Bright-field images were then collected every week and analyzed to assess visual degradation. The results indicate that tubular membranes made with 5, 10, and 20% PAK3-RGDS retained their tubular shape and were stable in PBS solution for up to 4 weeks, comparable to control (0% PAK3-RGDS) (Figure S2). Tubular membranes made with 50 and 70% PAK3-RGDS disintegrated after 1 week in PBS (Figure S2). Constructs made with 100% PAK3-RGDS failed to form a tubular membrane. Steric hindrance can influence formation and stability of self-assembling systems, which has been observed in self-assembling cyclic peptides, where bulky brush conformations of poly(ethylene glycol) macromolecules inhibited the assembly process.^36^ We suggest that for higher concentrations of PAK3-RGDS (above 20%), steric effects can distort the assembly process and result in poor tubular membrane stability. Additionally, tubular membranes made with up to 20% PAK3-RGDS exhibited a multilayered nanofibrous microarchitecture typical of the EL-PAK3 system.^30^ Based on the stability study results, subsequent biocompatibility experiments were conducted using tubular membranes made with 0, 5, 10, and 20% PAK3-RGDS (Figures 4A and 5A).

#### Cell Morphology on **EL-RGDS**/PAK3/PAK3-RGDS and **EL-noRGDS**/PAK3/PAK3-RGDS Tubular Membranes

We first investigated the effect on cell morphology of RGDS when present on the EL and PA molecules. mADSCs were seeded on both luminal (PA-rich) and abluminal (EL-rich) sides (Figure 1D) of the tubular membranes in a serum-free media. Cells were incubated for 4 h, washed to remove nonadherent cells, and incubated in full media (DMEM with 20% FBS) for additional 20 h. Constructs were then fixed for SEM imaging following standard protocols (as described in Methods). mADSCs grown on EL-RGDS/PAK3/PAK3-RGDS tubular membranes made with 5, 10, and 20% PAK3-RGDS exhibited a spread morphology with extensive lamellipodia on both luminal and abluminal sides, similarly to mADSCs grown on the abluminal side of the control tubular membranes (0% PAK3-RGDS) (Figure 4B,C). In contrast, cells grown on the luminal side of the control tubular membrane (0% PAK3-RGDS) (Figure 4C) exhibited more rounded morphologies. To better identify the influence of PAK3-RGDS, we then looked at the cell morphology of mADSCs grown on tubular membranes fabricated with EL-noRGDS. We observed that mADSCs grown on both the luminal and abluminal sides of 5 and 10% PAK3-RGDS of the EL-noRGDS/PAK3/PAK3-RGDS tubular membranes exhibited adherent morphology with some extended processes. However, cell shape was mostly triangular with limited spreading (Figure 5B,C), similarly to the control tubular membranes (0% PAK3-RGDS). We suggest that triangular cell shape might result from limited availability of RGDS sites, provided only by the PAK3-RGDS molecules in the EL-noRGDS/PAK3/PAK3-RGDS tubular membranes. When the concentration of PAK3-RGDS was increased to 20%, cell spreading increased on both luminal and abluminal sides (Figure 5B,C). It has been shown that spacing of RGDS epitopes influences cell morphology, for instance, larger spacing between RGDS epitopes resulted in poor spreading, elongated shape, and extended filopodia.^37^ Our cell morphology studies indicate that the presentation density of the RGDS epitope embedded on PA molecules influences cell spreading and morphology, which corresponds to previously reported literature;^38^ however, the influence of RGDS presentation density still needs to be clarified when it comes to cell adhesion.

#### Cell Adhesion on **EL-RGDS**/PAK3/PAK3-RGDS and **EL-noRGDS**/PAK3/PAK3-RGDS Tubular Membranes

We then looked at the effect of RGDS present or not in both EL and PA molecules on cell adhesion. Tubular membranes were prepared in the same way as described above, followed by fixing and staining with DAPI. Confocal microscopy observations indicated higher numbers of mADSCs growing on EL-RGDS/PAK3/PAK3-RGDS tubular membranes containing 5 and 10% of PAK3-RGDS compared to tubular membranes made with 0% (control) or 20% PAK3-RGDS (Figure 4D). Similar results were also observed for EL-noRGDS/PAK3/PAK3-RGDS tubular membranes (Figure 5D), suggesting that the incorporation of PAK3-RGDS has an effect on cell adhesion. These findings were further corroborated by quantitative analysis of fluorescent micrographs (Figures 4E and 5E), which revealed that cell adhesion was significantly higher in tubular membranes made with 5% PAK3-RGDS for both EL-RGDS/PAK3/PAK3-RGDS and EL-noRGDS/PAK3/PAK3-RGDS. Massia and Hubbell first showed that increases in surface concentration of RGD resulted in significant increases in adhesion of human foreskin fibroblast cells.^39^ Similar results were obtained by Webber et al., who also observed a rapid decrease of cell adhesion at higher concentrations of PA-RGDS, which was attributed to epitope crowding and saturation.^40^ Effects of epitope crowding and supramolecular packing were also investigated by Storrie et al.,^32^ who reported enhanced cell adhesion of 3T3 fibroblasts to a nanofibrous self-assembled PA material with lower packing of a bioactive epitope. In light of these studies, we suggest that the positive effect of PAK3-RGDS on cell adhesion in our study depends on supramolecular packing of RDGS epitopes and subsequent epitope mobility, which in turn affects signal accessibility. In conclusion, our results indicate significantly higher cell adhesion on tubular membranes made with 5% PAK3-RGDS for both EL-noRGDS/PAK3/PAK3-RGDS and EL-RGDS/PAK3/PAK3-RGDS membranes. However, differences between luminal and abluminal sides of the tubular membranes were not observed. These results highlight the importance of epitope dynamics and accessibility to cell signaling.

#### Cell Adhesion on Luminal versus Abluminal Sides of **EL-RGDS**/PAK3/PAK3-RGDS and **EL-noRGDS**/PAK3/PAK3-RGDS Tubular Membranes

We then investigated how incorporation of PAK3-RGDS would influence cell adhesion on the luminal side versus abluminal side of the tubular membranes (Figures 4F and 5F). Due to the presence of the RGDS epitope in the EL-RGDS molecule, cells attach preferentially on the abluminal side of the tubular membrane (EL-rich) compared to the luminal side (PA-rich).^30^ mADSCs were seeded on both luminal (PA-rich) and abluminal (EL-rich) sides (Figure 1D) of the tubular membranes in serum-free media to isolate the effect of the RGDS epitope. Cells were incubated for 4 h, washed to remove nonadherent cells, and incubated in full media (DMEM with 20% FBS) for an additional 20 h. Constructs were then fixed for SEM imaging following standard protocols (as described in Methods). Quantitative analysis of cell numbers was obtained from SEM micrographs. We observed a significant increase in cell density on the luminal side of the EL-RGDS/PAK3/PAK3-RGDS tubular membranes containing 5 and 10% PAK3-RGDS compared to the control (0% PAK3-RGDS). In contrast to the EL-RGDS/PAK3/PAK3-RGDS system, a significant increase in cell density was observed on the abluminal side of the EL-noRGDS/PAK3/PAK3-RGDS tubular membranes containing 5 and 10% of PAK3-RGDS compared to the control (0% PAK3-RGDS) (Figure 5F). These results are in agreement with the adhesion study (Figures 4E and 5E), where we observed that RGDS epitope spacing, epitope dynamics, and accessibility as well as effects from epitope crowding seem to influence mADSC adhesion to both luminal and abluminal sides of the tubular membranes.

#### Cell Metabolic Activity on **EL-RGDS**/PAK3/PAK3-RGDS and **EL-noRGDS**/PAK3/PAK3-RGDS Tubular Membranes

To further identify the effect of RGDS distribution on cell metabolic activity, an Alamar Blue assay was carried out on multiple time points over 3 days of cell culture (Figures 4G and 5G). The results revealed that the metabolic activity of cells grown on both EL-RGDS/PAK3/PAK3-RGDS and EL-noRGDS/PAK3/PAK3-RGDS tubular membranes containing 5 and 10% PAK3-RGDS is significantly increased compared to 20% PAK3-RGDS membranes at 34 and 58 h. These results indicate that addition of PAK3-RGDS (5% and 10%) has a positive effect on the metabolic activity of the mADSCs compared to tubular membranes with increased density of the PAK3-RGDS signal (20%). In addition to bioactive ligand spacing, integrin clustering and subsequent formation of stable focal adhesions have been shown to be key for efficient cell adhesion, spreading, and viability.^41,42^ Huang et al.^43^ observed that presence of at least three integrins per cluster favors maximum adhesion, whereas Schvartzman et al.^44^ reported a dramatic increase in spreading efficiency when at least four epitope sites were spaced within 60 nm or less, with no dependence on global density. We observed less cell spreading in conditions where there was less RGDS epitope (ELP-noRGDS/PAK3/PAK3-RGDS with 0, 5, and 10% of PAK3-RGDS). This result suggests that lower concentrations of RGDS may lead to lower integrin clustering, inhibiting the formation of focal adhesions and consequently leading to lower cell adhesion. Confirmation of this hypothesis would require further experimentation focused on identifying the precise localization of RGDS epitopes, which is beyond the scope of the current study.

In conclusion, by incorporating PAK3-RGDS into the EL/PAK3 system, it is possible to tailor cell adhesion and its localization on the luminal and abluminal sides of the coassembled tubular membranes. This anisotropic epitope distribution within the constructs results in differences in morphology and metabolic activity of mADSCs grown on the tubular membranes, opening the opportunity to generate biohybrid self-assembling constructs with selective cell distribution and behavior. More cell adhesion was found in 5% PAK3-RGDS of both EL-RGDS and EL-noRGDS tubular membranes, while better spreading was found in both 5 and 10% PAK3-RGDS of EL-RGDS tubular membranes as well as in 20% PAK3-RGDS of EL-noRGDS tubular membrane. Furthermore, increased metabolic activity was found in 5 and 10% PAK3-RGDS of both EL-RGDS and EL-noRGDS tubular membranes.

## Conclusions

To more accurately mimic biological processes, it is vital to develop innovative methods that enable the design of materials with tunable composition and structure from the molecular scale and up to the macroscale. Multicomponent self-assembly offers opportunities to engineer biomaterials in such a manner, facilitating incorporation of multiple signaling, tunability of structure, and communication with cells. In this study, we report an array of precise molecular modifications in PA and EL molecules that can be used to generate EL/PA coassembled materials with tailored structure and bioactivity. First, modification of molecular composition of PAs may be used to design EL/PA systems with specific mechanical properties and architecture. Second, use of bioactive PAs may enable generation of such biomaterials with precise biocompatibility and bioactivity profiles. Taken together, our study demonstrates that design of molecular composition of both PA and EL is paramount for optimization of biomaterial properties, such as hierarchical structure, construct stability, and signaling that improves the capacity to communicate with cells. Key advantages of the EL/PA coassembled materials include ease of fabrication, tunability of PA and/or EL composition, and the ability to incorporate other signals or molecules into the system. On the other hand, disadvantages are also present and include difficulty in reproducibility, a highly anisotropic microstructure, and sensitivity to ionic environments, which prevents membrane formation in physiological conditions.

## Supporting Information Available

Additional experimental details, materials, and methods, including proof of PA purity (PDF)
