# Supplementary material for: Peptide–Protein Coassemblies into Hierarchical and Bioactive Tubular Membranes
Source: Biomacromolecules. 2023 Jan 25;24(10):4419–29. doi: 10.1021/acs.biomac.2c01095 (PMC10565817; doi:10.1021/acs.biomac.2c01095)
Supplement: Supplementary file 1 — bm2c01095_si_001.pdf [file bm2c01095_si_001.pdf]

Supplementary Information

Peptide-Protein Coassemblies into Hierarchical and Bioactive Tubular Membranes

Anna Majkowska[1-3], Karla E. Inostroza-Brito[3], Mariel Gonzalez[3], Carlos Redondo-Gómez[2,3], Alistair Rice[4], Jose Carlos Rodriguez-Cabello[5], Armando E. Del Rio Hernandez[4], Alvaro Mata[2,3,6,7,8]*

1. William Harvey Research Institute, Queen Mary University of London, London, EC1M 6BQ, UK.
2. Institute of Bioengineering, Queen Mary University of London, London, E1 4NS, UK.
3. School of Engineering and Materials Science, Queen Mary University of London, London, E1 4NS, UK.
4. Department of Bioengineering, Imperial College London, London, SW7 2AZ, UK.
5. BIOFORGE Group, University of Valladolid, CIBER-BBN, 47011 Valladolid, Spain.
6. School of Pharmacy, University of Nottingham, Nottingham, NG7 2RD, UK.
7. Biodiscovery Institute, University of Nottingham, Nottingham, NG7 2RD, UK.
8. Department of Chemical and Environmental Engineering, University of Nottingham, Nottingham, NG7 2RD, UK.

Corresponding Author:

Prof. Alvaro Mata

School of Pharmacy

Department of Chemical and Environmental Engineering

Biodiscovery Institute

University of Nottingham

University Park

Nottingham, NG7 2RD

Phone: +44 (0)115 7494098

Email: a.mata@nottingham.ac.uk

Figure S1. PAs purity and structural conformation characterized by reverse phase HPLC and electrospray ionization mass spectrometry.

Figure S2. Stability of the EL/PAK3/PAK3-RGDS membranes in a PBS 1X solution. Bright field micrographs of EL/PAK3/PAK3-RGDS membranes (top view) before cross-linking, after cross-linking and after submerging in a PBS 1X solution at RT. Images were taken every week for up to 4 weeks. Level of membrane degradation is representative of stability.

Figure S1.

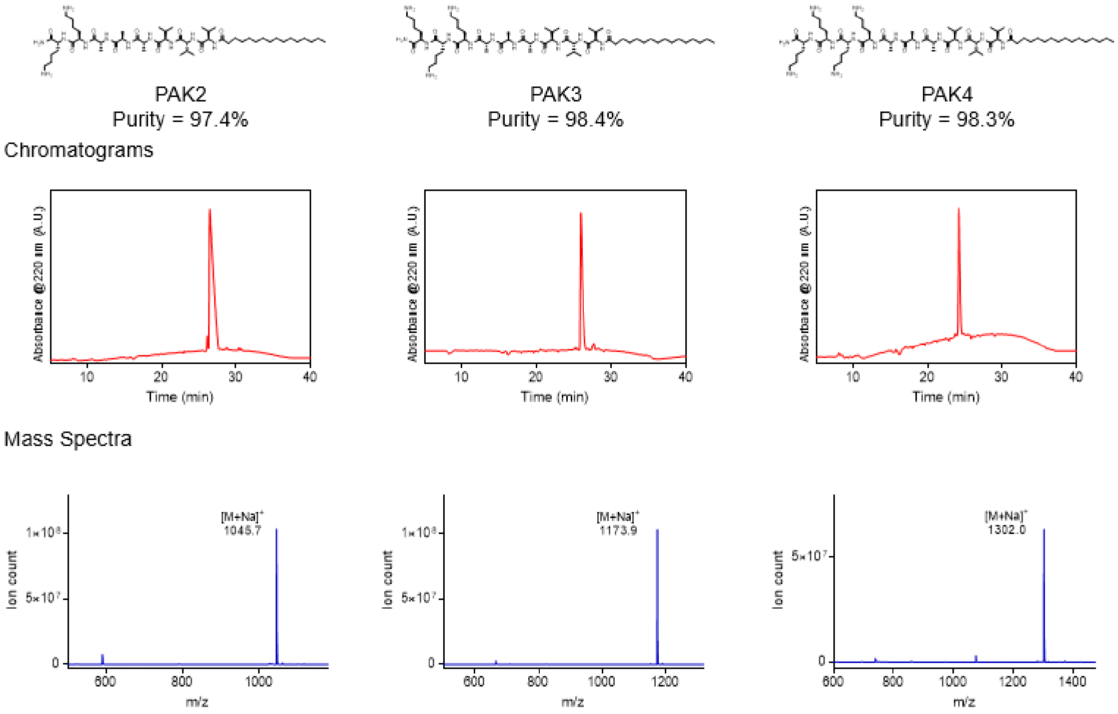
PAK2
Purity = 97.4%
PAK3
Purity = 98.4%
PAK4
Purity = 98.3%
Chromatograms
Absorbance @220 nm (A.U.)
Time (min)
10
20
30
40
Mass Spectra
Ion count
m/z
[M+Na]+
1045.7
1173.9
1302.0
600
800
1000
1200
1400

Figure S2.

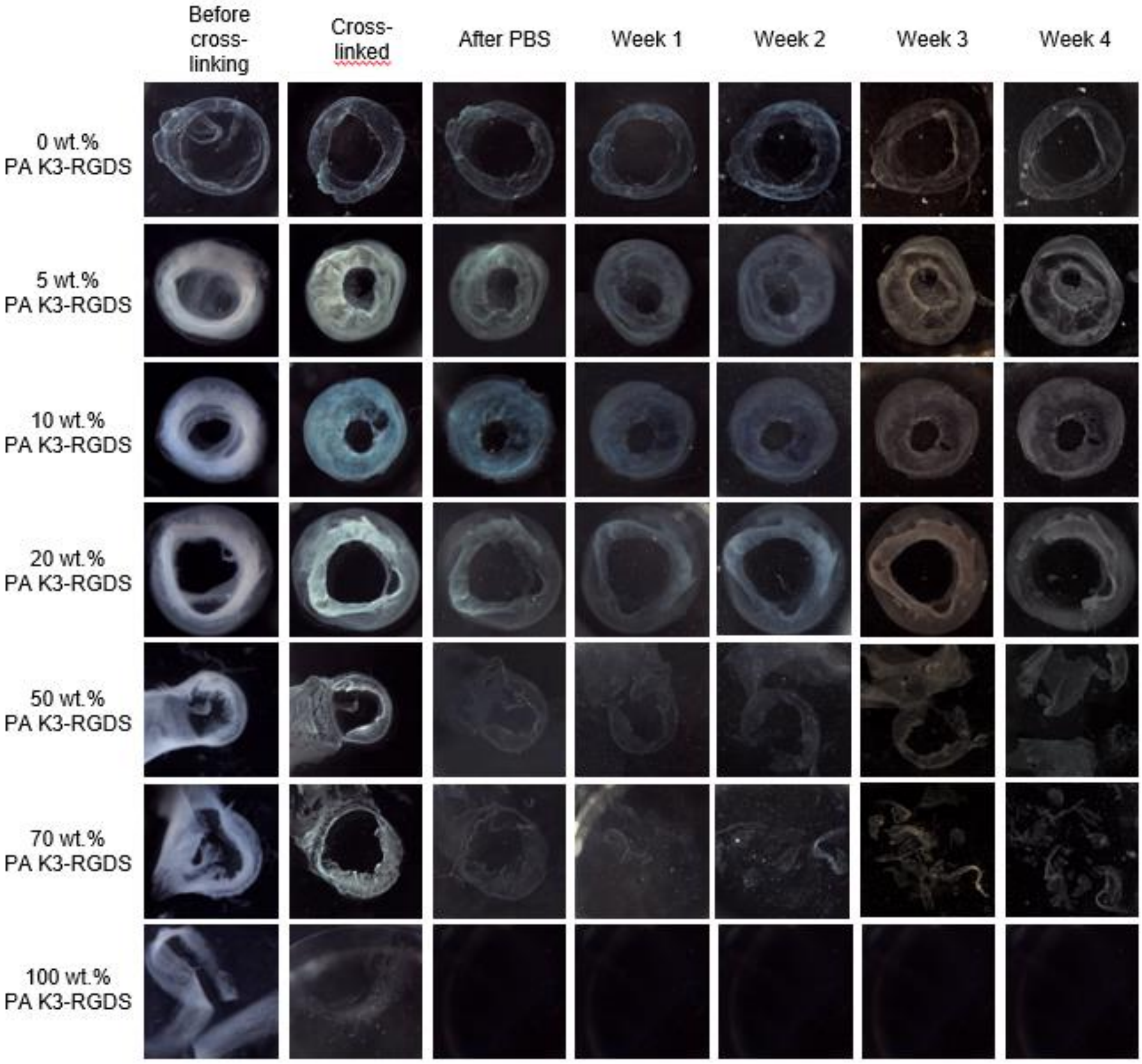

Before cross-linking
Cross-linked
After PBS
Week 1
Week 2
Week 3
Week 4
0 wt.% PA K3-RGDS
5 wt.% PA K3-RGDS
10 wt.% PA K3-RGDS
20 wt.% PA K3-RGDS
50 wt.% PA K3-RGDS
70 wt.% PA K3-RGDS
100 wt.% PA K3-RGDS
